# Association of Lipids with Oxidative Stress Biomarkers in Subclinical Hypothyroidism

**DOI:** 10.1155/2012/856359

**Published:** 2012-11-29

**Authors:** Adriana Santi, Marta M. M. F. Duarte, Charlene C. de Menezes, Vania Lucia Loro

**Affiliations:** Programa de Pós-Graduação em Bioquímica Toxicológica, Departamento de Química, Centro de Ciências Naturais e Exatas, Universidade Federal de Santa Maria, 97105-900 Santa Maria, RS, Brazil

## Abstract

*Objective*. The aim of the present study was to evaluate the oxidative stress biomarkers in patients with subclinical hypothyroidism (*n* = 20) and health controls (*n* = 20). *Subjects and Methods*. Total cholesterol (TC), triglycerides (TGs), low-density lipoprotein-cholesterol (LDL-C), high-density lipoprotein cholesterol (HDL-C), thiobarbituric acid reactive substances (TBARSs), catalase (CAT), superoxide dismutase (SOD), and arylesterase (ARE) were analyzed. *Results*. TC, LDL-C, TBARS, and CAT were higher in subclinical hypothyroidism patients, whereas SOD did not change. Arylesterase activity was significantly lower in the SH group, compared with the control group. Correlation analyses revealed the association of lipids (TC and LDL-C) with both oxidative stress biomarkers and thyrotropin (TSH). Thyroid hormones were correlated only with triglyceride levels. In addition, TSH was significantly correlated with TBARS, CAT, and SOD. However, no significant correlations were observed after controlling TC levels. *Conclusions*. We found that SH patients are under increased oxidative stress manifested by reduced ARE activity and elevated lipoperoxidation and CAT activity. Secondary hypercholesterolemia to thyroid dysfunction and not hypothyroidism *per se* appears to be associated with oxidative stress in subclinical hypothyroidism.

## 1. Introduction

Subclinical hypothyroidism (SH), defined as an elevated serum thyroid stimulating hormone (TSH) level associated with serum thyroid hormone concentrations within the reference range, is found in 4–10% of individuals from Western populations [1, 2]. Patients with hypothyroidism have an increased risk of developing atherosclerosis, and the subclinical stage is also considered a risk factor for this disease [3, 4]. Some investigators have found this connection to be attributed to increased levels of total cholesterol (TC), low-density lipoprotein cholesterol (LDL-C), and apolipoprotein (apo) B [5, 6], whereas others did not observe any significant differences [7, 8].

Thyroid hormones are associated with the oxidative and antioxidative status of the organism. Depression of metabolism due to hypothyroidism has been reported to decrease oxidant production and thus protects tissues against oxidant damage [9, 10]. However, data on the oxidative status of hypothyroidism are limited and controversial [11–13].

Lipid peroxidation (LPO) is a free radical chain reaction, which is triggered by hydroxyl radical and leads to membrane break. It facilitates the alteration in the protein structure and function and promotes generation of free radicals (FRs) [14]. LPO is reported to be high in hyperlipidaemia, which is a consistent biochemical feature in hypothyroidism [15]. A study has shown that LPO in subclinical hypothyroid patients was similar to that in normal controls [16], while another study found increased LPO in hypothyroid patients [12].

The biological oxidative effects of free radicals on lipids, proteins, and DNA are controlled by a spectrum of antioxidants. Enzymatic protection against reactive oxygen species (ROS) and the breakdown products of peroxidized lipids and oxidized protein and DNA are provided by several enzyme systems such as superoxide dismutase (SOD) and catalase (CAT) [17]. SOD catalyzes the dismutation of the superoxide anion into hydrogen peroxide (H_2_O_2_), which is then deactivated to water (H_2_O) by catalase or glutathione peroxidase (GPx) [18, 19].

High-density lipoproteins (HDLs) inhibit atherosclerosis development mainly by inducing reverse cholesterol transport [20]. However, other antiatherogenic effects of HDL have been reported, because of their apolipoprotein A-I (apo-AI) and paraoxonase 1 (PON1) content [21]. Arylesterase (AE), one of the enzymatic activities of paraoxonase-1, is known to play a protective role against peroxidation of LDL and other lipoproteins [22].

Given the high prevalence of SH in the general population, it is important to establish whether these alterations of thyroid function entail an oxidative stress and cardiovascular risk. Thus, the aim of this study was to assess the oxidative stress biomarkers and investigated their relation with lipid parameters in subjects with hypothyroidism.

## 2. Subjects and Methods

### 2.1. Subjects

Forty adult subjects from clinical laboratory LABIMED, Santa Maria, RS, Brazil were recruited for the present study. They were then classified into two groups-control group: 20 healthy subjects (47.20 ± 11.73 years) and the subclinical hypothyroidism (SH) group: 20 subjects newly diagnosed (49.12 ± 10.85 years). SH was defined as an elevated thyrotropin (TSH) (>4.5 mIU/L) and normal free thyroxine (FT4) level (8.7–22.6 nmol/L) [23]. Exclusion criteria were (1) lipid-lowering drugs, (2) antioxidant vitamin supplements, (3) acetylsalicylic acid, (4) antihistamines, (5) antihypertensive, (6) exposure to high-iodine condition, (7) smokers, (8) alcoholics, (9) pregnant, (10) hormone replacement therapy, (11) diabetes mellitus, and (12) acute, chronic, or malignant diseases. All subjects gave written informed consent to participate in the study. The protocol was approved by the Human Ethics Committee of the Federal University of Santa Maria (no. 23081.016996/2008).

### 2.2. Sample Collection

Blood samples were collected after 12 h overnight fasting by venous puncture into gray and red top Vacutainers (BD Diagnostics, Plymouth, UK) tubes. The samples were centrifuged for 15 min at 2500 ×g, and aliquots of serum were kept at −20°C for maximum of 4 weeks. An aliquot of whole blood was collected into sodium citrate (3.2%) and diluted 1 : 10 in saline solution for measurement of CAT and SOD activities. 

### 2.3. Thyroid Profile

Thyroid profile was assessed by estimation of serums TSH, T3, and fT4 that were measured by chemiluminescent immunometric assay on IMMULITE 2000 (Siemens Healthcare Diagnostics, Los Angeles, USA). Detection limits for TSH was 0.004–14.000 mIU/L, FT4 were 3.9–77.2 pmol/L, and T3 was 0.29 nmol/L. 

### 2.4. Lipid Profile

Serum total cholesterol (TC) and triglycerides (TG) concentrations were measured using standard enzymatic methods by use of Ortho-Clinical Diagnostics reagents on the fully automated analyzer (Vitros 950 dry chemistry system; Johnson & Johnson, Rochester, NY, USA). High-density lipoprotein cholesterol was measured in the supernatant plasma after the precipitation of apolipoprotein B-containing lipoproteins with dextran sulfate and magnesium chloride as previously described [24]. Low-density lipoprotein cholesterol (LDL-C) was estimated with the Friedewald equation [25]. 

### 2.5. Thiobarbituric Acid Reactive Substances Levels

Serum thiobarbituric acid reactive substances (TBARSs) were measured according to the modified method of Jentzsch et al. [26]. Serum was added to a reaction mixture containing 1% orthophosphoric acid, an alkaline solution of thiobarbituricacid-TBA, followed by heating for 45 min at 95°C. After cooling, samples and standards of malondialdehyde (0.03 mM) were read at 532 MDA/mL.

### 2.6. Catalase Activity

 Whole blood catalase (CAT) activity was determined by the method of Aebi [27] by measuring the rate of decomposition of H_2_O_2_ at 240 nm. An aliquot of blood was homogenized in potassium phosphate buffer, pH 7.0. The spectrophotometric determination was initiated by the addition of sample into an aqueous solution of hydrogen peroxide 0.3 mol/L. The change in absorbance at 240 nm was measured for 2 min. CAT activity was calculated using the molar extinction coefficient (0.0436 cm^2^/*μ*mol), and results were expressed as U/g Hb. 

### 2.7. Superoxide Dismutase Activity

Whole blood superoxide dismutase activity was measured as described by McCord and Fridovich [28]. In this method, SOD present in the sample competes with the detection system for superoxide anion. A unit of SOD is defined as the amount of enzyme that inhibits the rate of adrenalin oxidation by 50%. Adrenalin oxidation leads to the formation of the colored product, adrenochrome, which is detected spectrophotometrically. SOD activity is determined by measuring the rate of adrenochrome formation, observed at 480 nm, in a reaction medium containing glycine-NaOH (50 mM, pH 10.0) and adrenalin (1 mM). Basal measurements to calibrate the assay were performed in a reaction medium containing 1 mL of glycine-NaOH (50 mM, pH 10.0) and 17 *μ*L of adrenalin (1 mM). This was used to determine the concentration in samples. The results were expressed as U/mg Hb. 

### 2.8. Arylesterase Activity

Serum arylesterase activity was measured using phenylacetate (Sigma Co, London, UK) as the substrate. The phenol formed after the addition of a 40-fold diluted serum sample was measured spectrophotometrically at 270 nm following an established procedure [29]. Enzymatic activity was calculated from the molar absorptivity coefficient of the produced phenol, 1310 M^−1 ^cm^−1^. One unit of arylesterase activity was defined as 1 *μ*mol phenol generated/min under the above conditions and expressed as U/L serum ([15], view record in scopus).

### 2.9. Hemoglobin Determination

Hemoglobin concentrations were measured in whole blood with a Pentra 120 analyzer (ABX, Montpellier, France). The results were expressed as g/dL. 

### 2.10. Statistical Analysis

Data are presented as mean and standard deviation (SD). The nonparametric Mann-Whitney *U*-test was used to compare differences between groups. Spearman correlation was assessed to evaluate the correlations between the variables. Partial correlations were performed to control the associations between variables for total cholesterol levels. Statistical significance was assumed at *P* < 0.05. Data were analyzed using SPSS version 11.0 software (SPSS Inc., Chicago, IL, USA).

## 3. Results 

**Figure 1:**
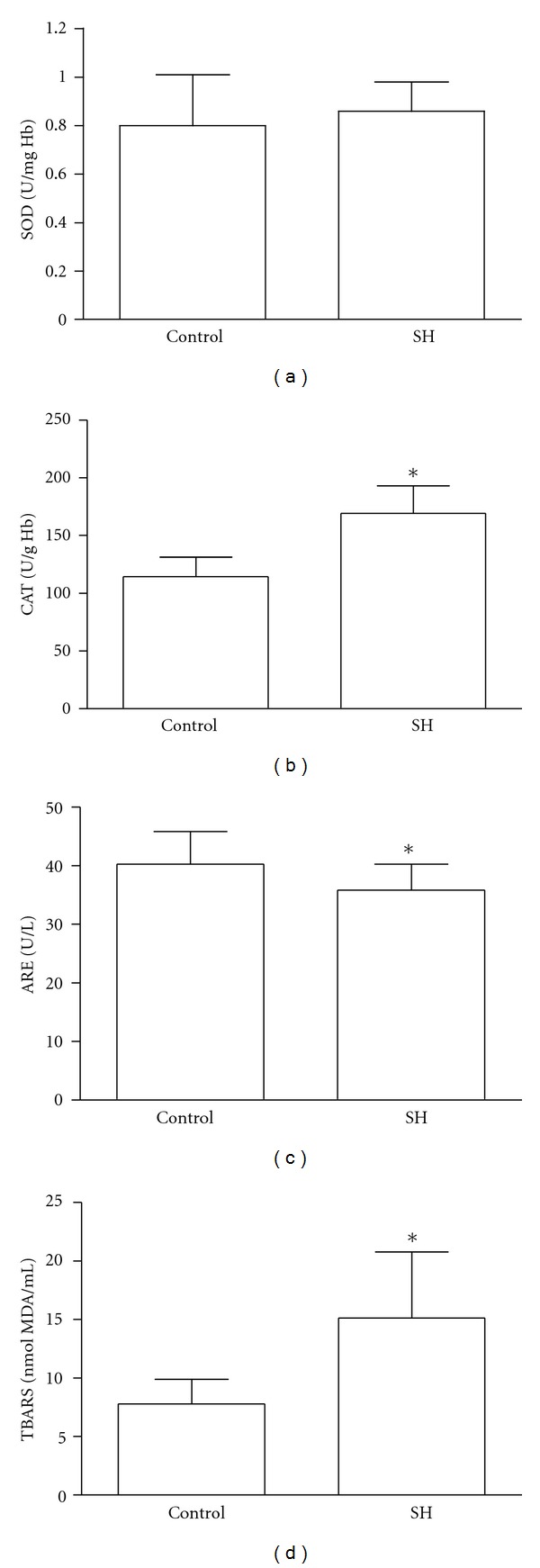
The values of SOD (a), CAT (b), ARE (c), and TBARS (d) in control and subclinical hypothyroidism (SH) groups. *P < 0.05.

There were no significant differences in age and body mass index (BMI) between groups. SH patients had significantly higher TSH, TC, LDL-C, and TC/HDL ratio with FT4 normal range than the control group (Table 1). In SH group TBARS and CAT were significantly higher than controls, while SOD did not change, as shown in Figure 1. 

Arylesterase activity was significantly lower in the group with SH, compared with the control group (Figure 1). ARE did not show any correlation with thyroid hormones, lipids and oxidative stress biomarkers. 

We observed a positive correlation between TC and TBARS (*r* = 0.757, *P* < 0.0001), TC and CAT (*r* = 0.650, *P* < 0.0001), LDL and TBARS (*r* = 0.812, *P* < 0.0001), LDL and CAT (*r* = 0.644, *P* < 0.0001), and LDL and SOD (*r* = 0.540, *P* < 0.001), as shown in Table 2. 

The correlations between TSH, T3, and fT4 with oxidative stress biomarkers are shown in Table 3. TSH was significantly associated with TBARS and CAT (*r* = 0.734, *P* = 0.000; *r* = 0.499, *P* = 0.004, resp.). However, no significant correlation was observed after controlling for TC levels. 

## 4. Discussion 

In the present study we have demonstrated that patients with subclinical hypothyroidism had altered lipid profiles, reduced ARE activity, increased lipid peroxidation, and induction of enzymatic defense when compared with control subjects. Hypercholesterolemia is a common feature in hypothyroidism since thyroid hormones upregulate LDL-receptor expression [30]. In a substantial number of studies, TC and/or LDL-C seem to be elevated in SH compared with controls [31–33]. In this respect, our results showed that subjects with SH had significantly higher levels of TC, LDL-C, TG, and TC/HDL-C ratio thus displaying a more atherogenic lipid profile when compared with healthy individuals. 

The level of lipid profiles is influenced by many factors. The present research has shown that thyroid hormones change the lipid profiles. Thyroid hormones may stimulate hydroxymethylglutaryl coenzyme A (HMG CoA), the key enzyme of cholesterol biosynthesis, and induce an increased synthesis of cholesterol. Additionally, the LDL-C receptor gene contains a thyroid hormone responsive element (TRE) that could allow triiodothyronine (T3) to modulate the gene expression of the LDL-C receptor resulting in an increase of LDL-C receptor synthesis. Thyroid hormones and their function are low in target tissue in SH, and researchers conjectured that SH influences lipid profiles by the above-mentioned mechanism [34, 35]. We report here a positive correlation between TSH and total cholesterol and LDL fraction as well as thyroid hormones (T3 and FT4) showing correlation with triglyceride levels. TSH was also associated with deleterious changes in serum lipids, particularly HDL-C, LDL-C, and the ratio of LDL-C to HDL-C as suggested by recent investigations [36–38].

Thyroid dysfunctions increase LPO reactions and ROS as documented by recent studies [39, 40]. LPO is an autocatalytic mechanism leading to oxidative destruction of cellular membranes [41]. Such destruction can lead to cell death and to the production of toxic and reactive aldehyde metabolites called free radicals, where malondialdehyde (MDA) is the most important. It is known that ROS would lead to oxidative damage of biological macro10molecules, including lipids, proteins, and DNA [10, 42]. We observed increased concentrations of TBARS in the circulation of SH patients. Moreover, TBARS was correlated with TSH, TC, and LDL cholesterol. However, after controlling TC levels, the association between TSH and TBARS was not significant suggesting that increased LPO could be attributed to lipid levels in hypothyroid status. Similar results were found by Nanda et al. (2008), where a significant correlation between TSH and MDA was lost after nullifying the effects of each of the coronary lipid risk factors among hypothyroid subjects [15].

Free radical-scavenging enzymes such as SOD and CAT are the first line of cellular defense against oxidative injury, decomposing O_2_^−^ and H_2_O_2_ before interacting to form a more reactive hydroxyl radical (OH). These enzymes protect the red cells against O_2_- and H_2_O_2_-mediated lipid peroxidation [19]. We have observed an increased activity of CAT in the SH group. In addition, our study shows an association between lipid parameters (CT and LDL) and CAT or SOD activities. Recently, Duarte et al. (2010) demonstrated that CAT was significantly higher in subjects with hypercholesterolemia [43]. On the other hand, some studies have reported no changes in CAT activity in hypothyroid patients [15, 44]. We also observed that the associations between TSH and TBARS, CAT, and SOD were lost when lipids (cholesterol) were annulled. Therefore, oxidative stress is likely to be potentially related to the secondary hypercholesterolemia to thyroid dysfunction and not directly to thyroid hormone levels in subclinical hypothyroidism. This result is consistent with our previous study showing that hypercholesterolemia has a stronger influence on the development of oxidative stress in overt hypothyroid (OH) patients [45].

It is supposed that LDL particles can be protected from free radical-induced oxidation by an HDL-linked enzyme, paraoxonase 1 (PON1). PON1 is found in tissues such as liver, kidney, intestine, and also serum [46]. It may possess antiatherogenic and anti-inflammatory properties, resulting from its ability to destroy modified phospholipids and to prevent cumulation of oxidized lipids in lipoproteins [47]. We found lower PON1 arylesterase activity in SH patients than controls, suggesting oxidative stress. Similar results were found by other researchers [48, 49]. In addition, epidemiological evidence demonstrates that low PON1 activity is associated with increased risk of cardiovascular events [50] and is an independent risk factor for cardiovascular disease [51].

In conclusion, our study shows an increase in the oxidative stress biomarkers in the circulation of patients with subclinical hypothyroidism. Oxidative stress biomarkers seem to be associated with secondary hypercholesterolemia to hypothyroidism, whereas hypothyroidism *per se *does not cause oxidative stress in SH patients. On the other hand, high-plasma lipids can be considered as an oxidation substrate for the oxidative stress [52]. Thus, we suggest monitoring of oxidant/antioxidant status and lipid levels in SH patients, because we have found associations between serum TSH levels and serum lipids levels, showing thyroid dysfunction influence on lipid metabolism and consequently on oxidant/antioxidant status in these patients. However, further studies are necessary to evaluate a larger series of patients, with a longer duration of subclinical hypothyroidism.

## Figures and Tables

**Table 1:** Clinical and laboratory data of study participants.

	Control	Subclinical hypothyroidism
*n *	20	20
Age (years)	47.20 ± 11.73	49.12 ± 10.85
Male (%)	50	50
BMI (Kg/m^2^)	21.30 ± 3.65	23.70 ± 3.20
TC (mmol/L)	4.28 ± 0.37	6.42 ± 0.84*
HDL (mmol/L)	1.51 ± 0.27	1.01 ± 0.19
LDL (mmol/L)	2.05 ± 0.43	4.61 ± 0.95*
TG (mmol/L)	1.72 ± 0.40	2.03 ± 0.69
TC/HDL	0.09 ± 0.03	0.16 ± 0.05*
TSH (mIU/L)	1.71 ± 0.78	11.62 ± 2.33*
T3 (nmol/L)	1.26 ± 0.23	1.15 ± 0.70
fT4 (pmol/L)	18.70 ± 5.54	19.09 ± 5.67

Data are expressed as mean ± SD. *P < 0.001. BMI: body mass index; TC: total cholesterol; HDL: high-density lipoprotein; LDL: low-density lipoprotein; TG: triglyceride; TSH: thyroid-stimulating hormone; T3: triiodothyronine; fT4: free thyroxine.

**Table 2:** Correlation analyses between oxidative stress biomarkers and lipid parameters in subclinical hypothyroidism and controls subjects.

	TBARS	CAT	SOD
	nmol MDA/mL	U/g Hb	U/mg Hb
TC (mmol/L)	0.757**	0.650**	0.209
HDL (mmol/L)	−0.302	−0.268	−0.258
LDL (mmol/L)	0.812**	0.644**	0.540*
TG (mmol/L)	0.113	0.433	0.333

*P < 0.001; **P < 0.0001. TC: total cholesterol; HDL: high-density lipoprotein; LDL: low-density lipoprotein; TG: triglyceride; TBARS: thiobarbituric acid reactive substances; CAT: catalase; SOD: superoxide dismutase.

**Table 3:** Correlations of serums TSH, T3, and fT4 with oxidative stress biomarkers in the whole population before and after controlling for total cholesterol (TC) levels.

Biomarkers	TSH				T3				fT4			
	Before		After		Before		After		Before		After	
	*r*	*P*	*r*	*P*	*r*	*P*	*r*	*P*	*r*	*P*	*r*	*P*
TBARS, nmol MDA/mL	0.734	0.000	0.149	0.497	−0.137	0.522	0.034	0.875	−0.008	0.969	0.217	0.319
CAT, U/g Hb	0.499	0.004	0.036	0.870	−0.278	0.124	−0.153	0.485	−0.269	0.137	−0.121	0.580
SOD, U/mg Hb	0.330	0.065	0.061	0.781	−0.106	0.566	0.084	0.702	0.014	0.938	0.169	0.440

*P* < 0.05 was considered statistically significant. TSH: thyroid-stimulating hormone; T3: triiodothyronine; fT4: free thyroxine; TBARS: thiobarbituric acid reactive substances; CAT: catalase; SOD: superoxide dismutase.
